# Right thyroid lobe agenesis and left thyroid colloid benign nodule discovered incidentally in female with breast carcinoma receiving chemotherapy for multiple metastases: Case report and review of the literature

**DOI:** 10.1016/j.ijscr.2023.108154

**Published:** 2023-04-11

**Authors:** Mohamed S. Al Hassan, Walid El Ansari, Nourelhuda Issa, Adham Darweesh, Abdelrahman Abdelaal

**Affiliations:** a Department of General Surgery, Hamad General Hospital, Doha, Qatar; b Department of Surgery, Hamad General Hospital, Doha, Qatar; c College of Medicine, Qatar University, Doha, Qatar; d Weill Cornell Medicine – Qatar, Doha, Qatar; e Department of Clinical Imaging, Hamad General Hospital, Doha, Qatar

**Keywords:** Thyroid, Hemiagenesis, Breast carcinoma, Metastasis, Colloid, Nodule, Qatar, Incidental

## Abstract

**Introduction and importance:**

Thyroid hemiagenesis (THA) is the failure of embryologic development of a lobe of the thyroid gland and is a rare anomaly of uncertain incidence. The left lobe is more commonly absent than the right lobe. It is discovered incidentally during investigations.

**Case presentation:**

A 48 year old Egyptian female presented at the thyroid surgery clinic at our institution to follow up after a nodule left thyroid lobe accidently discovered on positron emission tomography (PET) scan undertaken to follow up on bone metastasis of breast cancer which was surgically removed about 14 years ago.

**Clinical discussion:**

The patient looked clinically well with no scar in the anterior of the neck, no palpable thyroid nodules, and no lymphadenopathy. Ultrasound imaging of the neck revealed absent right thyroid lobe tissue and a nodule was noted at the upper pole of the left thyroid. Laboratory tests unremarkable, with TSH (2.14 mIU/L), and FT4 (12.4 pmol/L) within normal range. Fine needle aspiration and cytology of the thyroid nodule revealed atypia of undetermined significance.

**Conclusion:**

THA is rare and right THA is even rarer. It is usually asymptomatic, and diagnosis is mostly incidental while investigating symptoms due to pathology of the other thyroid lobe or any of the parathyroid glands. In much rarer circumstances, right THA might be discovered when investigating conditions not related to the thyroid or parathyroid glands years after the initial pathology as in the current case. Etiology is inconclusive but genetic factors could play a role. No treatment is required if no symptoms are present.

## Highlights

• Thyroid hemiagenesis (THA) is rare and right THA is even rarer
• Diagnosis is mostly incidental, usually because of symptoms due to pathology of the other thyroid lobe or parathyroid glands
• Rarely, right THA might be discovered when investigating conditions not related to thyroid or parathyroid glands.
• This could be more than a decade later after the initial pathology, which in the present case was left breast cancer
• No treatment is required if no symptoms are present.

## 1 Introduction

The thyroid is one of the largest glands weighing approximately 15–20 g in normal adults [1]. The two hormones it secretes influence almost every organ system in the body and play important roles in organ development and in homeostatic control of essential physiological mechanisms such as body growth and energy expenditure [1], [2]. Thyroid hormone secretion is regulated via the hypothalamic-pituitary-thyroid axis [1].

Thyroid hemiagenesis (THA) is the failure of embryologic development of a lobe of the thyroid gland and is a rare anomaly of uncertain incidence [3]. The left lobe is absent in 80 % of cases, the right is in 20 % of cases (left to right hemiagenesis ratio of 4:1), and the isthmus in 50 % of patients [3]. Most reported cases are female with the left thyroid lobe absent [4]. Most cases are sporadic, however familiar clusters have been documented [5].

THA is usually associated with normal thyroid function [5], where patients have absent thyroid lobe, with contralateral lobe in euthyroid state without abnormalities [3]. Nevertheless, a range of pathologies have been observed in the remaining thyroid tissue in THA, including hyperthyroidism, multinodular goiter, hypothyroidism, benign adenoma, adenocarcinoma, and Grave's disease [3]. Most THA patients report no symptoms, and the condition is discovered incidentally during investigations or intraoperatively [5]. As for management, THA patients risk developing supplementary thyroid pathologies and may advantage from l-thyroxine treatment to lower the thyrotropin levels to those of the normal population [6]. Further research is required to observe if such interference early in life would stop development of associated thyroid problems [6].

We report a 48 Egyptian female with right thyroid hemiagenesis seen at our institution. She had mastectomy for a left primary breast cancer 13 years ago. She had no thyroid complaints, and ultrasound (US) imaging of the neck confirmed absent right thyroid lobe tissue. We report this case in line with the updated consensus-based surgical case report (SCARE) guidelines [7].

## 2 Presentation of case

A 48 year old Egyptian female presented at our institution on July 2018 to follow up a nodule in the left thyroid lobe that was accidently discovered in positron emission tomography (PET) scan being done as follow up for her breast cancer. She had complained of neck fullness and choking. There was no previous thyroid surgery and no family history of thyroid disease. She did not consume alcohol and was a non smoker. In 2009, she was diagnosed with left breast cancer in Egypt (stage T3 N2 M0), managed by mastectomy, axillary clearance, then by chemotherapy and radiotherapy. In November 2012, she presented at our institution in Doha with bone metastasis. She was started on hormonal therapy.

**Fig. 1:**
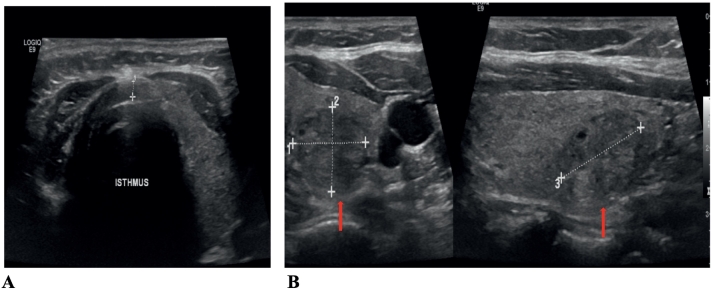
(A) Transverse ultrasound view of the neck at the thyroid gland level showing absent right thyroid lobe tissue; (B) transverse and longitudinal view of left lobe of thyroid gland showing solid heterogenous slightly hypoechoic nodule (red arrow).

Upon physical examination, the patient looked clinically well with no visible scar in the anterior aspect of the neck, no palpable thyroid nodules, and no lymphadenopathy. She was conscious and alert, there was no exophthalmos, and systems examination was unremarkable. Her laboratory tests were unremarkable, with TSH (2.14 mIU/L), and FT4 (12.4 pmol/L) within normal range. Ultrasound (US) imaging of the neck revealed absent right thyroid lobe tissue (Fig. 1). Left thyroid measures 17 mm with normal vascularity. An isoechoic complex nodule was noted at the upper pole (14 mm × 12 mm). There were no pathologic enlarged lymph nodes.

Because of previous history of metastasis from her breast cancer, FNA evaluation was advised and undertaken. Fine needle aspiration and cytology (FNAC) of the thyroid revealed atypia of undetermined significance (AUS). The case was discussed at our thyroid multi-disciplinary team (MDT) meeting, and the recommendation was to follow-up by US after 6 months.

The patient was followed up for 4 years with US neck undertaken on January 2019, and then February and July 2021. The US scans revealed slightly increased size of the left thyroid nodule and confirmed the absence of the right thyroid lobe. US neck in November 2021 showed a well-defined solid heterogenous predominantly mildly hypoechoic nodule (ACR TR4) in the lower left lobe measuring about 20 mm (previously 18 mm).

Fine needle aspiration and cytology (FNAC) were undertaken for the nodule on February 2021 and October 2022. Both revealed follicular cells and abundant colloid in a background of blood. There were no atypical or malignant cells seen. The patient had PET scan on September 2021 that showed stable appearance of hypermetabolic left thyroid lobe nodule without any significant interval change. Tracer uptake in the head and neck region was physiologic, and there was no nodal hypermetabolism or cervical adenopathy. There was no apparent abnormal cerebral FDG uptake. The patient also developed a liver nodule that was discovered during a PET scan on January 2021 that revealed a newly developed 1.7 cm metabolic diameter. There was fluorodeoxyglucose (FDG) accumulation in Segment VII of the liver close to diaphragmatic surface.

**Fig. 2:**
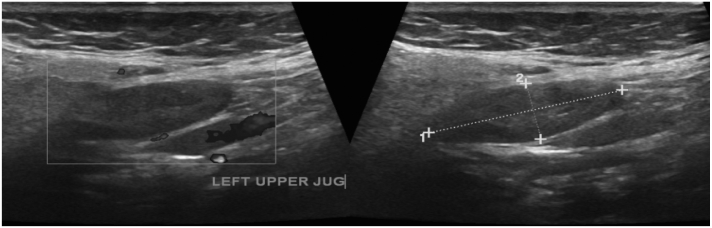
Ultrasound of the neck at upper jugular level showing mildly enlarged hypoechoic left upper jugular lymph node with absence of fatty hilum.

The patient also had US guided biopsy from the supraclavicular neck lymph nodes on February 2021 that revealed metastatic carcinoma with features suggestive of a breast primary (Fig. 2). Our recommended plan was to follow up the thyroid nodule with US and FNA to ensure there was no malignant cells.

## 3 Discussion

Thyroid Hemiagenesis (THA) is a rare congenital anomaly [5], and right THA is even rarer. We report a 48 year old Egyptian female with right THA discovered incidentally at our institution. We also report the findings of the literature review of right thyroid hemiagenesis case reports that we undertook, where we summarize the characteristics of the reported cases that were identified.

As for demography, our literature review (Table 1) shows that the age range of right-sided THA cases varied from 18 years [8] to 66 years [9]. However, many cases were observed in individuals between 30 and 58 years of age. Our patient's age was 48 years, falling within the age range reported by others [10], [11], [12], [13]. In terms of sex, THA is seen more often among females (female to male ratio 3:1) [9], [13], in support of our case who was a female. Table 1 also shows that females were more common, with a female to male ratio 5.5:1. THA, especially on the right side, may be influenced by sex-related factors similar to those in patients with an ectopic thyroid gland [13].

**Table 1:** Literature review: Summary of characteristics of right thyroid hemiagenesis case reports.

Study/year	Country	Age	Sex	Other pathology/abnormality	ETT	Diagnosis	Context of diagnosis	FH^a^	R PT pathology
Current study	Qatar	48	F	Breast cancer, metastasis to bone, LN, liver; colloid nodule in L T	No	PET, US	I; follow up of L BC	No	No
Khatri 1992 [14]	USA	41	F	L T lobe PA, which was nodular goiter 7 years ago	No	IS	I; follow up of mass in L T	**—**	No
Shibutani 1995 [15]	Japan	58	F	L lobe subacute thyroiditis	No	Tc-99m	I; painful mass in L lobe	No	No
Shaha 1997 [16]	USA	30	F	L T PC carcinoma	No	T scan	I; evaluating L T swelling	**—**	No
Bando 1999 [17]	Japan	42	F	Chronic autoimmune thyroiditis associated with HPT	No	Tc-99m	I; malaise, systemic edema, cold intolerance	No	No
Huang 2002 [10]	Taiwan	47	F	L T lobe PC	Yes, IH	US, Tc-99m	I; evaluating L T mass	**—**	No
Sakurai 2007 [11]	Japan	42	M	L lower PT adenoma	No	CT, Tc-99m	I; evaluating PHPT	**—**	No
Canani 2008 [18]	Italy	35	F	PC of thyroglossal duct cyst	No	US, MRI	I; evaluation of neck mass	**—**	No
Oruci 2009 [9]	Serbia	66	F	L T lobe PHPT, HT; R PT adenoma	No	Tc-99m, US	I; tumor in L T lobe	No	Adenoma
Velayutham 2013 [8]	India	18	M	Absent isthmus	Yes, SH & IH	US	I; swelling in front of neck	**—**	No
Wang 2014 [12]	China	49	F	L T MTC, absent isthmus	No	Tc-99m, US	I; routine medical checkup	No	No
Eroglu 2015 [19]	Turkey	27	F	R PT adenoma	No	Tc-99m	I; evaluating HPT	**—**	Adenoma
Gandla 2020 [20]	India	20	F	L T lobe PC	No	US, CT	I; solitary L T nodule	**—**	No
Kada 2021 [13]	Japan	48	F	PTC in all glands	No	CT	I; during follow up of GN	**—**	PTC

Due to space limitation, only the first author is cited; BC: breast cancer; CT: computed tomography; ETT: ectopic thyroid tissue; FH: family history; F: female; GN: glomerulonephritis; HPT hyperparathyroidism; HT: Hashimoto thyroiditis; I: incidental; IS: isotope scanning; IH: infrahyoid; L: left; M: male; MRI: magnetic resonance imaging; MTC medullary thyroid cancer; PA: papillary adenocarcinoma; PC papillary carcinoma; PET: positron emission tomography; PT parathyroid; PTC: parathyroid carcinoma; PTMC: papillary thyroid microcarcinoma; R: right; SH: suprahyoid; T: thyroid; Tc-99m: technetium 99mTc-methoxyisobutyl isonitrile (MIBI) scintigraphy; THA: thyroid hemiagenesis; US: ultrasound; **—**: no reported.

a Of thyroid disease.

In terms of geographical distribution of right-sided THA, Table 1 depicts that 4 of the14 cases we identified where in Japan (28.5 %). In a Japanese study of 151,374 male and 148,534 female subjects, THA was detected in 0.016 % and 0.027 % of the sample respectively [13]. In the same study, the prevalence of right THA (12 subjects) was significantly lower than that of left hemiagenesis (p < 0.001) [13]. Another study in Turkey reported THA prevalence of 0.25 % in an outpatient referral population and 0.025 % in a normal population [21]. Table 1 shows that THA is also observed in many other parts of the globe.

As regards to location, the left lobe is absent in 80 % of cases and the right lobe is absent in 20 % of cases [3]. Hence, THA is more commonly seen on the left side, with ratio 4:1 [9]. Systematic ultrasound evaluation confirmed the female predominance and higher incidence of left THA [22]. Hence, our literature review focused on the rarer cases of right THA for which we identified 14 cases. To the best of our knowledge, we are not aware of any similar cases published in the North Africa and Arabian Gulf region.

Pertaining to diagnosis, patients with THA are usually asymptomatic and generally discovered incidentally. Most published cases of THA generally and of right THA in particular have been observed incidentally after patients were examined for thyroid or parathyroid conditions [14], [19]. In agreement, Table 1 shows that all the cases were discovered incidentally when investigations were undertaken to investigate contralateral lobe pathology. Less frequently, THA is discovered when investigating a non-thyroid condition such as the current case, where the patient was being followed up for bone metastasis after breast cancer. Only then, a PET scan confirmed that the right thyroid lobe was absent. THA *per se* usually does not cause complaints by itself [21], and the first systematic ultrasound evaluation of THA in normal children confirmed a 0.2 % prevalence [22].

In connection with etiology, members of the PAX family are required for cell growth and differentiation in fetal tissues [23]. Consequently, *PAX8* participates in regulation of embryogenesis of the thyroid gland [23]. *PAX8* gene belongs to the paired-box (PAX) family of nine transcriptional factors (PAX1–9) [24]. It was observed in the single lobe of THA and it was not present in the other thyroid side [23]. However, others noted that the candidate gene *Pax8* may not be a key genetic factor in THA and that other genes may be involved [6].

In terms of associated pathologies with THA, Table 1 shows that 3 of the 14 cases had right side parathyroid pathologies, and all cases had left thyroid lobe pathology. A study of patients with thyroid disorders found 12 cases with thyroid hemiagenesis (0.25 %) [21].

As for management, none is required if patient is euthyroid. However, follow up and investigations are needed to discover any associated thyroid or parathyroid abnormalities that should then be managed accordingly.

## 4 Conclusion

THA is rare and right THA is even rarer. It is usually asymptomatic, and diagnosis is mostly incidental. In much rarer circumstances, THA might be discovered when investigating conditions not related to the thyroid or parathyroid glands, which in the present case, was left breast cancer. Etiology is inconclusive. No treatment is required if no symptoms are present. Further research to investigate the incidence of THA in general and particularly right THA are required, as well as the potential etiology of such cases.

## Consent

Written informed consent was obtained from the patient for publication of this case report and accompanying images. A copy of the written consent is available for review by the Editor-in-Chief of this journal on request.

## Provenance and peer review

Not commissioned, externally peer-reviewed.

## Ethical approval

Ethical approval was provided by the authors institution.

## Funding

None.

## Guarantor

Prof. Dr. Walid El Ansari.

## Research registration number

Not first in man.

## CRediT authorship contribution statement

**Mohamed S. Al Hassan**: data collection, data interpretation, writing the paper. **Walid El Ansari**: study concept, data interpretation, writing the paper, review & editing. **Nourelhuda Issa**: Writing - review & editing. **Adham Darweesh**: Imaging data preparation and interpretation, Writing - review & editing. **Abdelrahman Abdelaal**: study concept, Writing - review & editing. All authors read and approved the final version.

## Conflicts of interest

Nothing to declare.
